# AI avatars in education: effects on knowledge acquisition, academic motivation, and cognitive load

**DOI:** 10.3389/fpsyg.2026.1860755

**Published:** 2026-08-20

**Authors:** Elaheh Bakhtiari, Rossella Suriano, Giorgio Mario Grasso

**Affiliations:** NISC Lab, Department of Cognitive Science, University of Messina, Messina, Italy

**Keywords:** AI avatars, cognitive load, digital learning, knowledge acquisition, motivation

## Abstract

As digital learning environments increasingly incorporate artificial intelligence, empirical evidence regarding the educational effectiveness of AI-powered digital avatars remains limited and methodologically heterogeneous. This randomized matched-pair study examined whether learning with a human-like AI-avatar instructional system implemented in Unity 3D with Convai and OpenAI GPT-4o for real-time verbal interaction improves knowledge acquisition, academic motivation, and perceived cognitive load compared with autonomous internet-based learning. Forty-eight university students were included in the final analytic sample and were balanced across instructional condition and gender (AI-avatar: *n* = 24; control: *n* = 24; 12 males and 12 females in each condition). Participants completed an instructional task on introductory artificial intelligence and neural-network concepts, followed by a 20-item knowledge test, the Academic Motivation Scale, and a shortened Cognitive Load Scale. Separate 2 x 2 analyses of variance tested the effects of instructional condition and gender for each dependent variable. Reliability was satisfactory for the Academic Motivation Scale (pre-test Cronbach's *α* = 0.88; post-test *α* = 0.97) and the Cognitive Load Scale (*α* =.77). The AI-avatar group obtained higher knowledge-test scores than the control group (*M* = 10.63, *SD* = 2.39 vs. *M* = 8.92, *SD* = 3.11), *F*(1, 44) = 4.66, *p* = 0.036, partial *η*^2^ = 0.096. Academic motivation showed a more favorable pre-to-post trajectory in the AI-avatar condition (ΔSDI *M* = −1.15, *SD* = 3.67) than in the control condition (ΔSDI *M* = −4.36, *SD* = 3.70), *F*(1, 44) = 9.22, *p* = 0.004, partial *η*^2^ = 0.173; the unadjusted post-test SDI condition effect approached significance (*p* = 0.051), and an ANCOVA controlling for baseline motivation was significant (*p* = 0.006). Perceived cognitive load did not differ significantly between conditions, *F*(1, 44) = 0.16, *p* = 0.688. No significant gender main effects or condition-by-gender interactions emerged for the main outcomes. These findings suggest that AI-avatar-supported instruction may enhance knowledge acquisition and support motivational regulation, while evidence for cognitive-load reduction was not observed in the present data. The novelty of the study lies in its direct empirical comparison of next-generation, human-like AI-avatar learning support with conventional self-directed online learning, while also reporting outcome-specific analyses and limitations relevant to reproducibility.

## 1 Introduction

In the era of educational digitalization, tools based on artificial intelligence (AI) are redefining how students access, process, and assimilate knowledge (Herbert and Dołżycka, 2024; Suriano et al., 2025). Among the most significant innovations, digital avatars—with their advanced conversational interaction capabilities—offer personalized responses, adapt communication to the student's skill level, and facilitate an immersive learning experience (Fink et al., 2024; Johnson and Lester, 2016). Unlike other similar technologies limited to verbal or textual responses, digital avatars integrate visual and behavioral elements, such as facial expressions and vocal modulation, thus providing a more natural and engaging interaction (Alam and Mohanty, 2023; Lacković and Olteanu, 2026). In line with Bandura (1986) social-cognitive learning theory, which posits that the interactive environment can influence cognitive and emotional processes, the use of digital avatars could help create a dynamic educational context capable not only of increasing the interactivity of the learning experience but also of stimulating motivation and active participation among students (Fabio et al., 2025; Wu et al., 2024; Netland et al., 2025; Troussas et al., 2025).

In recent years, the effectiveness of virtual pedagogical agents in the context of online learning has been the subject of growing empirical interest. Evidence from meta-analyses and systematic reviews (Apoki et al., 2022; Castro-Alonso et al., 2021; Dai et al., 2022; Lane and Schroeder, 2022) indicates that the use of pedagogical agents is associated with a statistically significant improvement in learning performance compared to control conditions lacking such agents. However, the effect sizes observed are heterogeneous and influenced by moderating variables such as the duration of the intervention, the level of interactivity, and the visual and functional characteristics of the agents themselves. Recent attention has been particularly focused on AI-based agents designed to replicate human-like behaviors and communication modes through natural language, facial expressions, and gestures. According to the quantitative syntheses conducted by Dai and Ke (2022) and Dai et al. (2024), these advanced AI agents are significantly more effective than those without intelligent components, especially when characterized by an anthropomorphic appearance and synchronous audiovisual presentation. Avatars with human-like features appear to promote deeper cognitive processing of content, thanks to greater communicative salience and more efficient attentional orientation (Wang et al., 2018). Elements such as simulated eye contact, deictic gestures, and expressive tone of voice can serve as guiding signals, helping filter out irrelevant stimuli and focusing attention on central concepts. These signals reduce the burden of autonomous information selection and enable a more efficient use of available cognitive resources (Mayer, 2014; Dargue et al., 2019; Li et al., 2023). This focused attention helps minimize distractions from irrelevant information, thereby reducing perceived extraneous cognitive load—the mental effort spent processing unnecessary material. By highlighting essential elements and clarifying relationships between visuals and spoken explanations, deictic gestures make it easier for learners to integrate new information into working memory, which supports deeper understanding and better retention (Li et al., 2024; Beege et al., 2023).

This process aligns with the principles of Cognitive Load Theory (CLT; Sweller, 2011), which suggests that effective learning depends on the ability to manage and optimize limited cognitive resources. In this context, the introduction of a virtual pedagogical agent can function as an external guide, supporting the learner in the selection, organization, and integration of relevant information. Recent meta-analyses (Beege et al., 2023; Mohammad Yusoff et al., 2024) confirm that, when well-designed, virtual agents not only avoid overloading the learner but can enhance learning efficiency by acting as cognitive facilitators, freeing up mental resources allocable to deep understanding, critical analysis, or content generalization (Azevedo, 2005). Specifically, AI-based avatars can support this process in several ways. First, they can reduce *extraneous* cognitive load—generated by elements not directly related to the learning task—through personalized, sequential, and multimodal presentation of information, reducing the ambiguity and disorganization typical of autonomous internet searches. Second, these agents can optimize *essential* cognitive load by facilitating the processing of complex content through clear explanations, analogies, and immediate feedback, thus reducing the need for the user to independently construct complex cognitive schemas. Additionally, AI-powered agents can provide metacognitive support that aids learning regulation, such as controlling the pace of the lesson, allowing questions, or providing real-time clarifications (Chen et al., 2025). These metacognitive tools not only optimize the learning process but also increase the user's sense of control. The perception of being able to independently manage one's learning path reduces cognitive anxiety—a factor that can hinder learning—and promotes engagement (Shehzad et al., 2026; Teng and Wang, 2024). This is consistent with research highlighting how metacognitive regulation, facilitated by intelligent technologies, can enhance motivation and learning effectiveness (Ishak et al., 2025; Sui et al., 2024; Hicks, 2024).

Overall, previous research suggests that pedagogical agents and AI-supported instructional systems can positively influence learning performance, learner engagement, and motivation, particularly when they include interactive, multimodal, and anthropomorphic features. Meta-analytic evidence indicates that intelligent virtual agents may facilitate cognitive processing by guiding attention, reducing unnecessary cognitive effort, and enhancing learner interaction. However, findings remain inconsistent regarding the extent to which AI-powered digital avatars specifically influence key educational outcomes such as knowledge acquisition, academic motivation, and cognitive load. Moreover, much of the existing literature has focused on conventional virtual tutors or rule-based conversational agents rather than next-generation AI-driven avatars with advanced natural language and multimodal capabilities. These inconsistencies underscore the need for further empirical investigation.

Despite the growing interest, scientific literature still provides limited and conflicting evidence regarding the effectiveness of digital avatars in influencing crucial learning variables. Moreover, the avatars considered in these studies primarily rely on traditional conversational agents or fixed-rule virtual tutors developed through symbolic programming, rather than advanced linguistic models such as next-generation Neural Language Models (NLMs). Understanding how these tools compare to the traditional use of the internet in autonomous information search is essential for guiding future educational applications of artificial intelligence.

This study aims to fill this gap by comparatively analyzing the learning experiences of students interacting with an AI-based digital avatar versus those using autonomous online resource consultation. The goal is to investigate the effects of using a digital avatar on three fundamental dimensions of the learning process: knowledge acquisition, academic motivation, and perceived cognitive load. In response to these gaps, the present study examines the learning experiences of students interacting with an AI-powered digital avatar and those engaging in autonomous internet-based learning. Specifically, this study aims to evaluate whether AI-avatar-supported instruction produces measurable differences in (a) knowledge acquisition, (b) academic motivation, and (c) perceived cognitive load compared with conventional self-directed online information searching. Additionally, the study explores whether these effects vary according to participant gender. By examining both cognitive and motivational dimensions of learning, the present research seeks to provide empirical evidence regarding the educational effectiveness of AI-powered avatars and their potential role in technology-enhanced learning environments. Based on the existing literature, the following hypotheses are formulated:

**H1:** Students who learn by interacting with an AI-based digital avatar will acquire significantly more knowledge compared to those who study through autonomous internet search.
**H2:** Students who interact with the digital avatar will report significantly higher levels of academic motivation compared to the group that studies autonomously via the internet.
**H3:** Students who interact with the digital avatar will perceive significantly lower cognitive load compared to those who study autonomously via the internet.

## 2 Materials and methods

### 2.1 Participants

The sample consisted of 52 university students (age range: 18–35 years; *M =* 26.24; *SD =* 3.34) enrolled at the University of Messina, who voluntarily participated in the study from courses across different academic disciplines. Participants were randomly assigned to one of two experimental groups: (1) Internet-based Knowledge Acquisition (*n =* 24), in which learning occurred using the Internet, and (2) AI-Avatar Interaction (*n =* 24), in which learning was mediated by artificial intelligence-based avatars.

Randomization was conducted using a matched-pair procedure based on gender and intelligence quotient (IQ) to ensure initial group equivalence on these potentially confounding variables (Balzer et al., 2014; Sun et al., 2024). Gender distribution was balanced (24 males and 24 females), and all participants demonstrated cognitive functioning within the normal range, as assessed by Raven's Progressive Matrices (*M =* 100; *SD =* 15), consistent with normative population values (Murphy et al., 2023; Schinka and LaLone, 1997). One male participant was excluded from the analysis because his estimated IQ score, derived from the Raven's Progressive Matrices, was 83. According to the exclusion criterion adopted a priori and based on the recommendations of (Acosta Echavarria et al., 2022), participants with IQ scores in the borderline range were excluded to reduce the likelihood of including individuals with undetected intellectual impairment. Another male participant decided to not continue the experimental session, therefore a total of 4 participants were not considered, two females and two male, to keep the sample perfectly balanced. Table 1 presents the socio-demographic characteristics of the sample.

**Table 1:** Socio-demographic characteristics of the sample.

Variable		Experimental group *N*(%)	Control group *N*(%)
Age	*M*(*SD*)	25.88 (3.44)	26.60 (3.26)
Region	Iran	21 (84)	21 (84)
	Russia	1 (4)	0 (0)
	Italy	1 (4)	1 (4)
	Tunisia	1 (4)	0 (0)
	Bangladesh	1 (4)	1 (4)
	India	0 (0)	1 (4)
	Pakistan	0 (0)	1 (4)
Level of education	Undergraduate	24 (96)	20 (80)
	Graduate	1 (4)	5 (20)
Area of education	Cognitive science	13 (52)	10 (40)
	Political science	4 (16)	4 (16)
	Biology	0 (0)	1 (4)
	International mgmt.	0 (0)	2 (8)
	Geophysical science	1 (4)	2 (8)
	Medicine	1 (4)	1 (4)
	Data analysis	5 (20)	2 (8)
	Business management	1 (4)	1 (4)
	Computer science	0 (0)	2 (8)
Duration of English study (years)	< 1	4 (16)	7 (28)
	1–2	4 (16)	7 (28)
	3–5	7 (28)	6 (24)
	>5	10 (40)	5 (20)
Language course	IELTS	25 (100)	22 (88)
	TOEFL	0 (0)	3 (12)
Current English level	Fluent	3 (12)	2 (8)
	Advanced	14 (56)	9 (36)
	Intermediate	8 (32)	14 (56)
General AI information	Very poor	2 (8)	0 (0)
	Poor	5 (20)	7 (28)
	Fair	11 (44)	12 (48)
	Good	7 (28)	6 (24)

### 2.2 Procedure

The study was conducted in accordance with the ethical principles outlined in the Declaration of Helsinki and received approval from the Ethics Committee of the Cognitive Science Department of the University of Messina (COSPECS). Prior to the start of the experimental procedures, all participants provided written informed consent, voluntarily agreeing to take part in the research.

Participants were welcomed into the NISC (Neuro-Informatica e Scienze Cognitive) Laboratory of the University of Messina, which had been specifically prepared to ensure an optimal environment for concentration and comfort. An examiner provided them with the necessary materials to carry out the experiment. During all sessions subjects were left alone in the laboratory, to avoid distractions, and most sessions were video recorded for further analysis.

In a preliminary phase, participants were briefed on the scope of the research and were asked to complete a series of questionnaires designed to collect demographic data and assess various psychometric variables. Among the instruments administered was a test for the assessment of general cognitive abilities, followed by a questionnaire aimed at evaluating academic motivation. These sessions lasted approximately one hour each.

**Figure 1:**
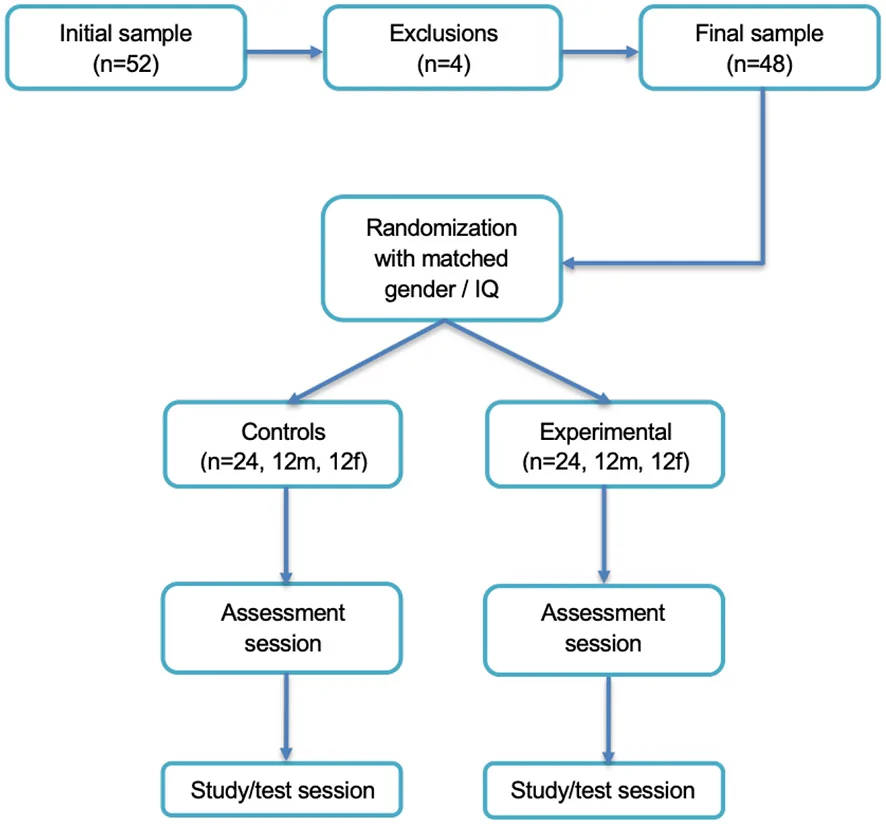
Block diagram of the experimental design and procedure followed during sessions with participants.

**Figure 2:**
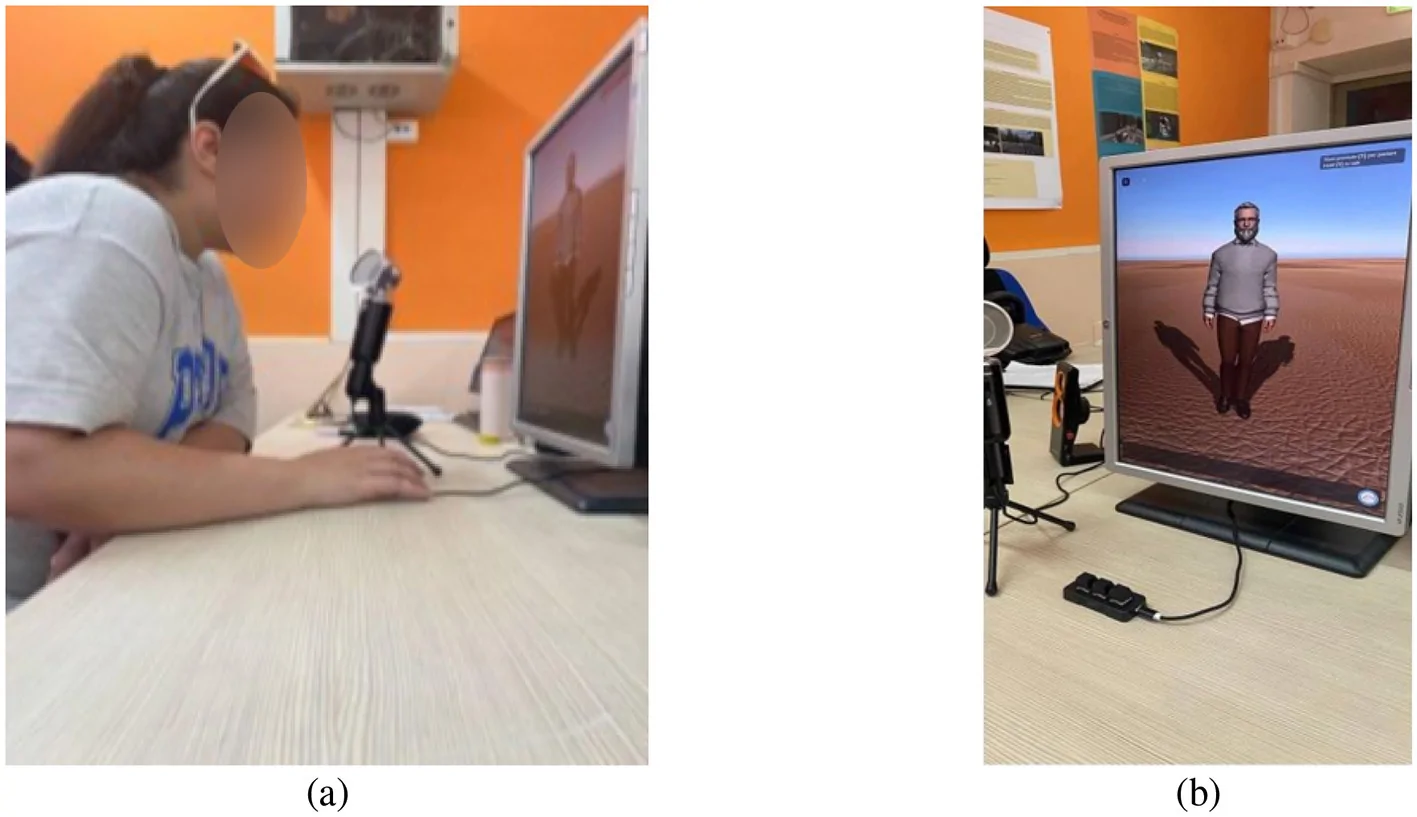
Participant engages with an AI avatar in the laboratory **(a)**, typical screen appearance of the AI avatar during experiments **(b)**

Subsequently, a second appointment was scheduled, during which participants took part in the experimental phase. In this phase, which lasted one hour, each individual interacted for 40 minutes with the material specific to their assigned group, and upon completion of the interaction, they completed a series of questionnaires aimed at assessing knowledge acquisition, academic motivation, and perceived cognitive load (Figure 1). Figure 2 illustrates the experimental learning environment in which participants interacted with the AI-powered digital avatar. The avatar was used to provide structured instructional guidance and facilitate participant engagement during the learning task. All participants completed the experiment under standardized conditions to ensure consistency across sessions. Specifically, they interacted with the avatar in the same environment, received identical instructions, were provided with the same initial prompt, completed the interaction within the same predefined duration, used the same hardware and software configuration - Intel i7 9700 Workstation with Windows 10, 64GB of RAM, 6TB of storage, GTX1080Ti GPU with Unity 3D ver. 2022.3.11f - and interacted with an identically configured avatar.

### 2.3 AI-avatar instructional system

The experimental condition used a screen-based, human-like three-dimensional AI-avatar instructional interface developed in the Unity 3D framework and implemented through the Convai commercial platform. The avatar was connected to the OpenAI GPT-4o large language model to enable real-time verbal interaction with participants. The system was voice-enabled and visually embodied: participants interacted orally with the avatar, received spoken responses in real time, and viewed synchronized mouth movements generated through lip-sync within Unity 3D. Gesture animations were also implemented to convey a more realistic and socially salient interaction. Accordingly, the intervention combined LLM-driven conversational guidance, speech-based interaction, visual embodiment, lip synchronization, and animated gesture cues.

The avatar was configured to provide instructional support within the same content domain studied by the control group. Its role was to explain, clarify, and organize the assigned introductory material on artificial intelligence and neural networks, while avoiding unrelated topics and avoiding provision of answers to the post-intervention assessment. Participants could ask questions verbally during the learning phase and receive concise explanations, examples, and clarifications through the avatar interface. This configuration was intended to operationalize the avatar as an embodied pedagogical agent rather than as a general-purpose search tool.

To standardize the intervention, the learning topic, content outline, task instructions, laboratory setting, and post-intervention assessment were kept constant across participants. Both conditions addressed the same topic: introductory concepts in artificial intelligence and neural networks, including definitions of AI, machine learning and deep learning, philosophical issues such as the Turing Test and Chinese Room argument, and basic neural-network concepts. The AI-avatar group received this support through real-time spoken interaction with the embodied avatar, whereas the control group studied the same domain through autonomous internet-based consultation.

### 2.4 Measurement

#### 2.4.1 Raven's progressive matrices (RPM)

The Raven's Progressive Matrices test (Raven, 1986, 2000) is a widely used tool for assessing cognitive abilities, primarily focused on measuring fluid intelligence. Its utility stems from its relative insensitivity to sociocultural factors such as environmental context and educational background. The test is structured as a sequence of diagrams or matrices composed of abstract geometric figures, each missing a specific element. The standard version is divided into five sets (A through E), each characterized by increasing levels of complexity, comprising a total of 60 items. Participants are required to identify the underlying pattern governing the arrangement of the figures and select the appropriate figure from the provided options.

#### 2.4.2 Academic motivation scale (AMS)

Academic motivation was assessed using the Academic Motivation Scale (Vallerand and Reid, 1984), a tool composed of 28 items that measures various dimensions of motivation within an educational context. The dimensions explored include: amotivation, external regulation, introjected regulation, identified regulation, intrinsic motivation for knowledge, intrinsic motivation for accomplishment, and intrinsic motivation for stimulation. Responses were evaluated on a 7-point Likert scale, with values ranging from 1 (*not at all true*) to 7 (*exactly true*). In line with Self-Determination Theory (Deci and Ryan, 1985), a Self-Determination Index (SDI) was calculated, providing a synthetic measure of the degree of self-determined motivation. SDI values range from −18 to +18, with higher scores reflecting a greater level of self-determined motivation. The scale exhibited high internal consistency, with Cronbach's *α* =.91 at pre-test and *α* =.97 at post-test.

#### 2.4.3 Cognitive load scale (CLS)

Perceived cognitive load was assessed using a shortened version of the Cognitive Load Scale (CLS; Krell, 2015), including six items in total: three for the intrinsic cognitive load dimension and three for the extraneous cognitive load dimension, in line with CLT (Sweller, 2011). Items ask participants to reflect on the complexity and clarity perceived during the task (e.g., “The questions were difficult to solve” [intrinsic]; “At times, I felt exhausted searching for the important information” [extraneous]). Responses are provided on a 5-point Likert scale (1 = *Strongly disagree* to 5 = *Strongly agree*). The scale demonstrated good reliability, as evidenced by high item-total correlations and McDonald's *ω* estimates (Li and Pengbo, 2024; Krell, 2015; Krell et al., 2022). In the present study, Cronbach's *α* =.79, indicating good internal consistency.

#### 2.4.4 Knowledge acquisition test

Knowledge acquisition was assessed through a researcher-developed multiple-choice assessment designed to evaluate participants' understanding of introductory concepts in General Artificial Intelligence and Neural Networks. The instructional content used in both experimental conditions was derived from educational materials developed by MinnaLearn in collaboration with the University of Helsinki through the *Elements of AI* initiative. The learning materials included approximately 21 pages of written content covering introductory AI topics, including the definition of artificial intelligence, related fields (e.g., machine learning, deep learning, and data science), philosophy of AI (including concepts such as the Turing Test and Chinese Room argument), and neural network fundamentals, such as neural structures, computational principles, and advanced neural network techniques. Participants in both groups studied the same educational material; however, the experimental group received instructional support through an AI-powered digital avatar, whereas the control group completed the learning task independently through autonomous internet-based consultation of the same content.

Following completion of the learning session, participants completed a 20-item multiple-choice knowledge assessment administered under standardized conditions without external assistance. Participants were allocated 20 min to complete the test. Each correct response was assigned one point, producing a total score ranging from 0 to 20, with higher scores indicating greater knowledge acquisition. Example assessment items included judgments regarding whether specific technologies or computational procedures (e.g., photo-editing functions, stock market prediction models) could be classified as forms of artificial intelligence. To minimize assessment bias, all participants completed the same evaluation procedure and scoring was based exclusively on objectively correct responses. As the assessment strategy evaluates objective knowledge rather than a latent psychological construct, traditional psychometric indices (e.g. internal consistency reliability) are not considered relevant for this type of assessment.

### 2.5 Statistical analysis

To evaluate the effects of instructional modality and participant gender on learning outcomes, a two-way analysis of variance (ANOVA) was conducted. The two independent variables included Instructional Condition—AI-Avatar Interaction (Experimental) vs. Internet-Based Search (Control)—and Gender (Male vs. Female). The analysis considered both the main effects of each factor and the interaction effect between them on key dependent variables: knowledge acquisition, academic motivation, and perceived cognitive load.

**Figure 3:**
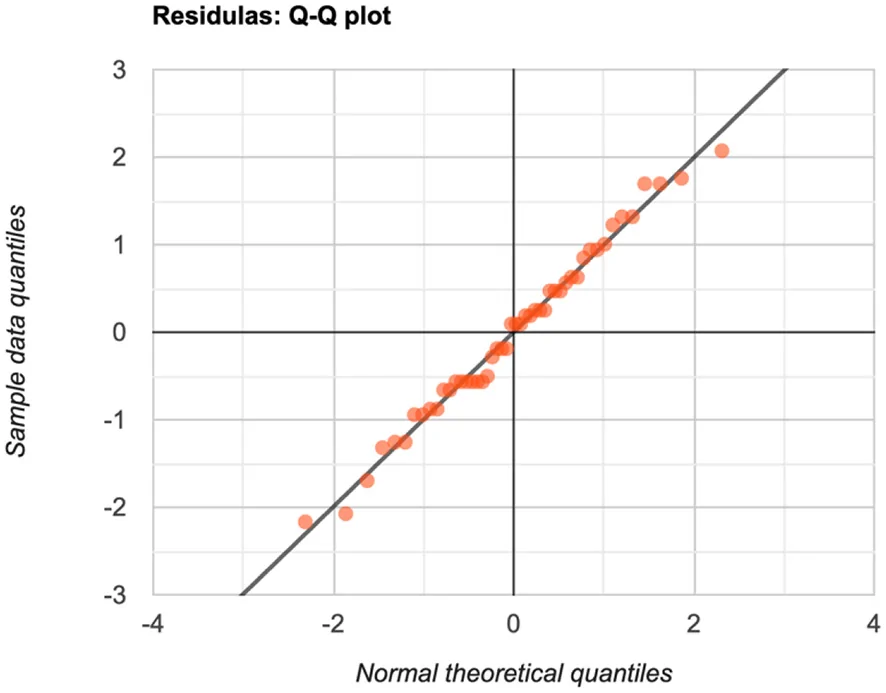
Q–Q plot illustrating the statistical soundness of the sample.

Before proceeding with the ANOVA, standard assumptions were tested. The data demonstrated normal distribution, as confirmed by the Shapiro–Wilk test (*p =* .52), and homogeneity of variance was visually inspected using Q–Q plots and residual diagnostics, showing no notable deviations (Figure 3). As illustrated in Figure 3, the Q–Q plot demonstrated that the observed values closely followed the expected normal distribution, supporting the assumption of normality required for ANOVA analysis. The significance threshold was set at *α =* .05 for all inferential tests. Where applicable, effect sizes were reported using partial eta squared (*η*^2^), and statistical power was calculated to determine the sensitivity of the test in detecting true effects. Post-hoc statistical power was calculated for each analysis to evaluate the sensitivity of the statistical tests. For all statistical analysis Python scripts have been implemented, using the “statsmodels” module for python.

## 3 Results

**Figure 4:**
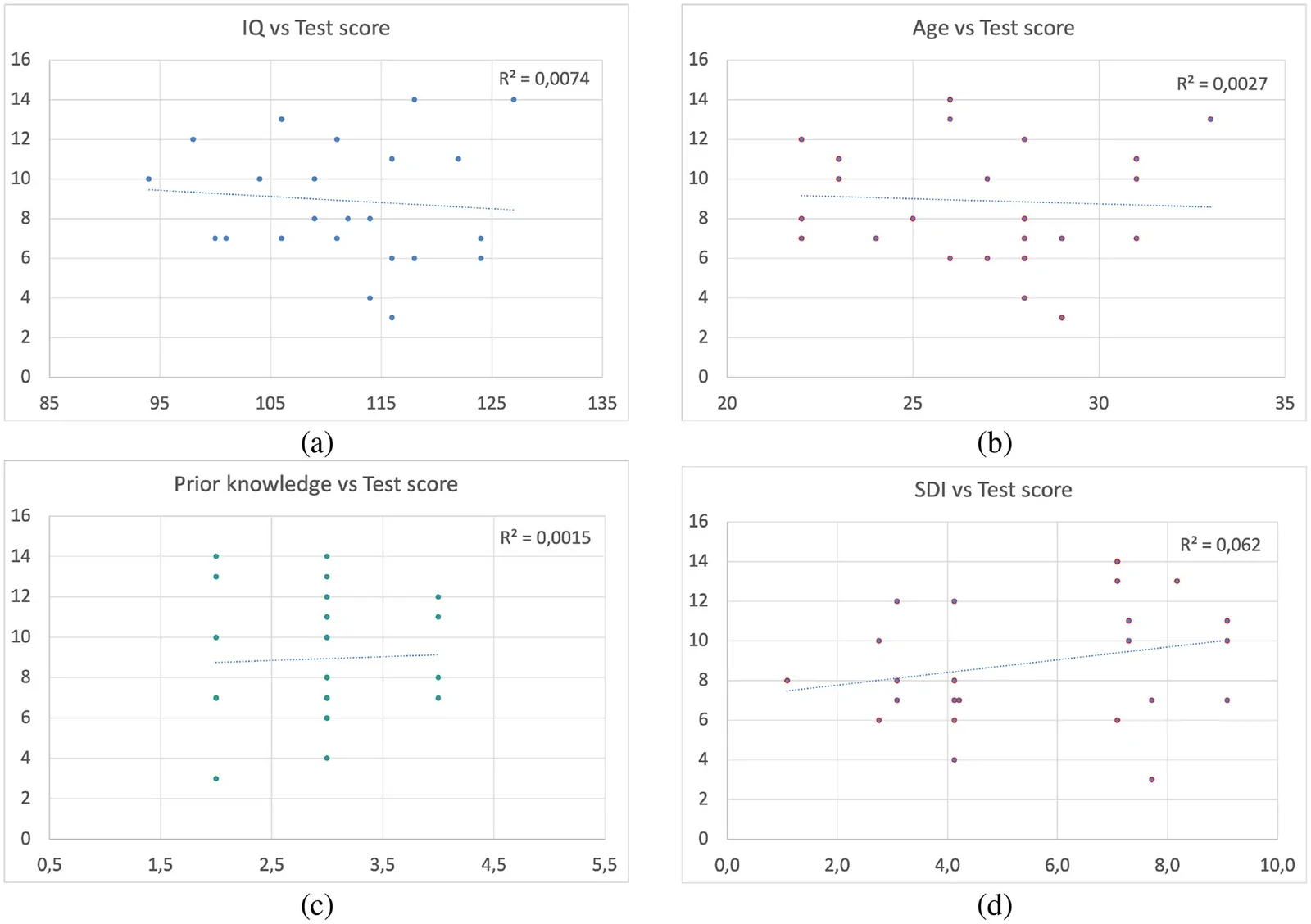
Correlation plots for control group. On the vertical axis the test score and on the horizontal axis the IQ **(a)**, the age **(b)**, the prior knowledge **(c)** and the SDI **(d)**.

**Figure 5:**
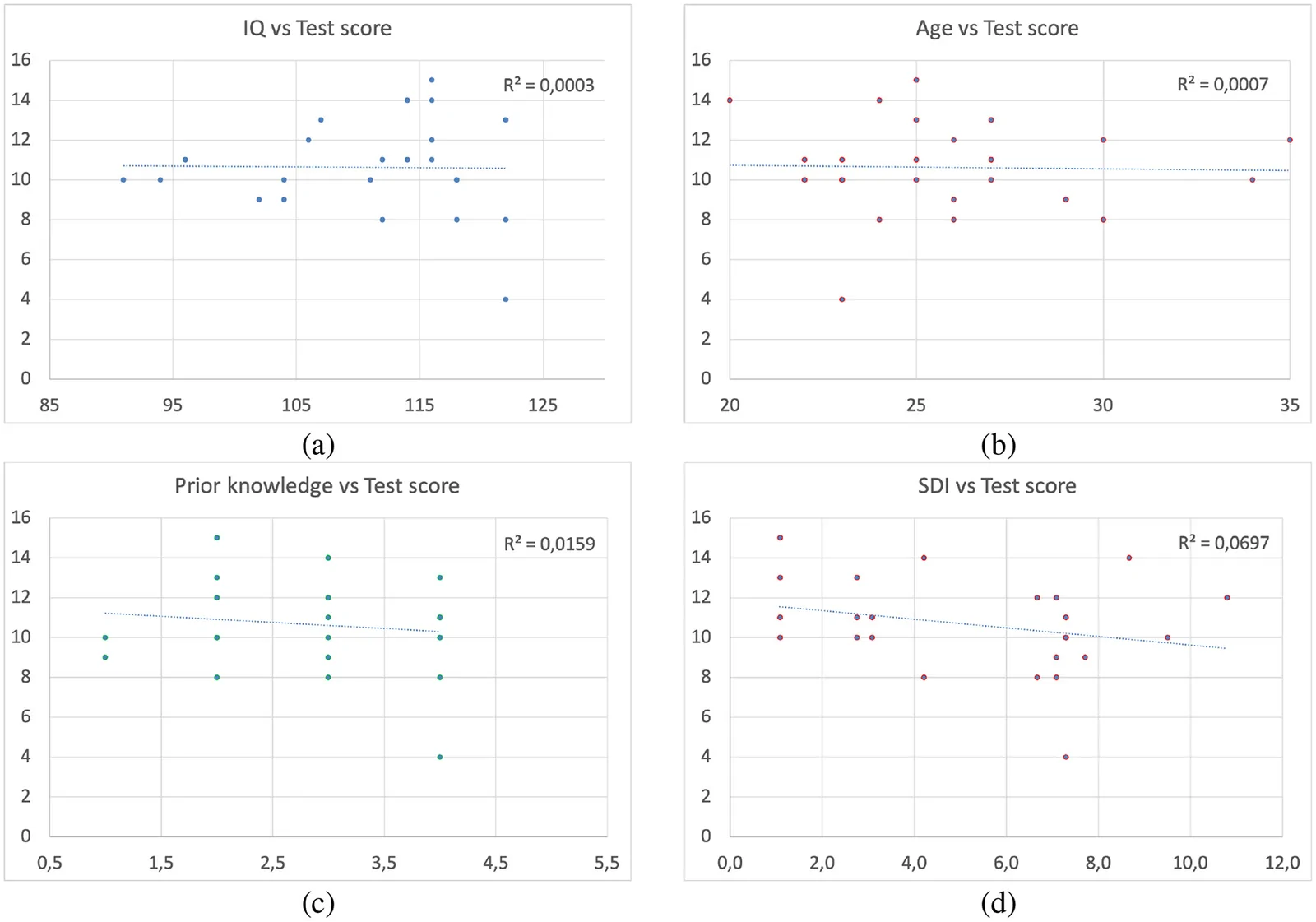
Correlation plots for experimental group.On the vertical axis the test score and on the horizontal axis the IQ **(a)**, the age **(b)**, the prior knowledge **(c)** and the SDI (**d)**.

Preliminarly, we explored potential associations between final test scores and individual difference variables, including IQ, initial academic motivation, prior knowledge, and age. No significant correlations were observed between these variables and learning outcomes. Scatterplots (Figures 4, 5) further illustrate the lack of systematic relationships, they illustrate the absence of meaningful relationships between individual difference variables (IQ, age, academic motivation, and prior knowledge) and final test performance in the control group, suggesting that learning outcomes were not substantially influenced by these participant characteristics.

The AI-avatar group obtained higher knowledge-test scores (*M* = 10.63, *SD* = 2.39) than the internet-based control group (*M* = 8.92, *SD* = 3.11). The condition effect was statistically significant, *F*(1, 44) = 4.66, *p* =.036, partial *η*^2^ =.096. The estimated condition difference was 1.71 points on the 0-20 test, 95% CI [0.09, 3.32], with Hedges' *g* = 0.61. The gender main effect and the condition-by-gender interaction were not significant. These results support H1.

Academic motivation was evaluated using the SDI. The unadjusted post-test SDI was higher in the AI-avatar condition (*M* = 4.27, *SD* = 3.98) than in the control condition (*M* = 1.86, *SD* = 4.41), but the two-way ANOVA condition effect narrowly missed the conventional significance threshold, *F*(1, 44) = 4.02, *p* =.051, partial *η*^2^ =.084. Because baseline motivation was available and differed by gender, the primary motivation analysis examined SDI change from pre-test to post-test. The AI-avatar group showed a significantly more favorable motivational change (*M* = −1.15, *SD* = 3.67) than the control group (*M* = −4.36, *SD* = 3.70), *F*(1, 44) = 9.22, *p* =.004, partial *η*^2^ =.173. The estimated condition difference in Delta SDI was 3.22, 95% CI [1.07, 5.36], Hedges' *g* = 0.86. An ANCOVA predicting post-test SDI while controlling for pre-test SDI also showed a significant condition effect, *F*(1, 43) = 8.27, *p* =.006. These results provide qualified support for H2: the AI-avatar condition was associated with better motivational maintenance or regulation, especially when baseline motivation was accounted for.

Mean cognitive load was similar in the AI-avatar condition (*M* = 3.08, *SD* = 0.50) and the control condition (*M = 3.18, SD* = 1.21). The condition effect was not significant, *F*(1, 44) = 0.16, *p* =.688, partial *η*^2^ =.004. The pairwise condition difference was -0.10, 95% CI [-0.65, 0.44], Hedges' *g* = −0.11. Variance heterogeneity was observed for cognitive load, Levene *p* =.002, but a Welch comparison was also non-significant, *p* =.699. The gender main effect and the condition-by-gender interaction were not significant. H3 was therefore not supported.

These findings suggest that, within our sample, performance differences on the final assessments were not driven by cognitive ability, prior knowledge, motivational levels, or age, thereby supporting the internal validity of the observed effects of the instructional conditions.

After confirming that performance was not influenced by individual difference variables, we assessed the impact of instructional modality on learning outcomes. The analysis yielded a statistically significant main effect of instructional modality on overall learning outcomes. Participants who engaged with the AI-based digital avatar demonstrated superior performance across dependent measures compared to those who learned via autonomous internet research. Specifically, the ANOVA revealed a statistically meaningful difference (*F*(1, 44) = 4.66, *p =* .036), with a medium effect size (*η*^2^ = 0.096) and high statistical power (0.923). These results indicate that interaction with the AI avatar had a notable and reliable positive impact on participants' academic engagement and understanding of the material.

In contrast, the main effect of gender was not statistically significant (*F*(1, 44) = 2.66, *p* =.110, *η*^2^ = 0.057), suggesting that male and female participants performed similarly regardless of the learning condition. Figure 5 shows no consistent associations between participant characteristics and test performance in the experimental group, further supporting the interpretation that observed learning differences were primarily associated with instructional condition rather than individual factors.

Furthermore, the analysis did not reveal a significant interaction effect between instructional condition and gender (*F*(1, 44) = 0.34, *p =* .566, *η*^2^ = 0.008), indicating that the instructional benefits of the AI-avatar were not differentially moderated by gender.

**Figure 6:**
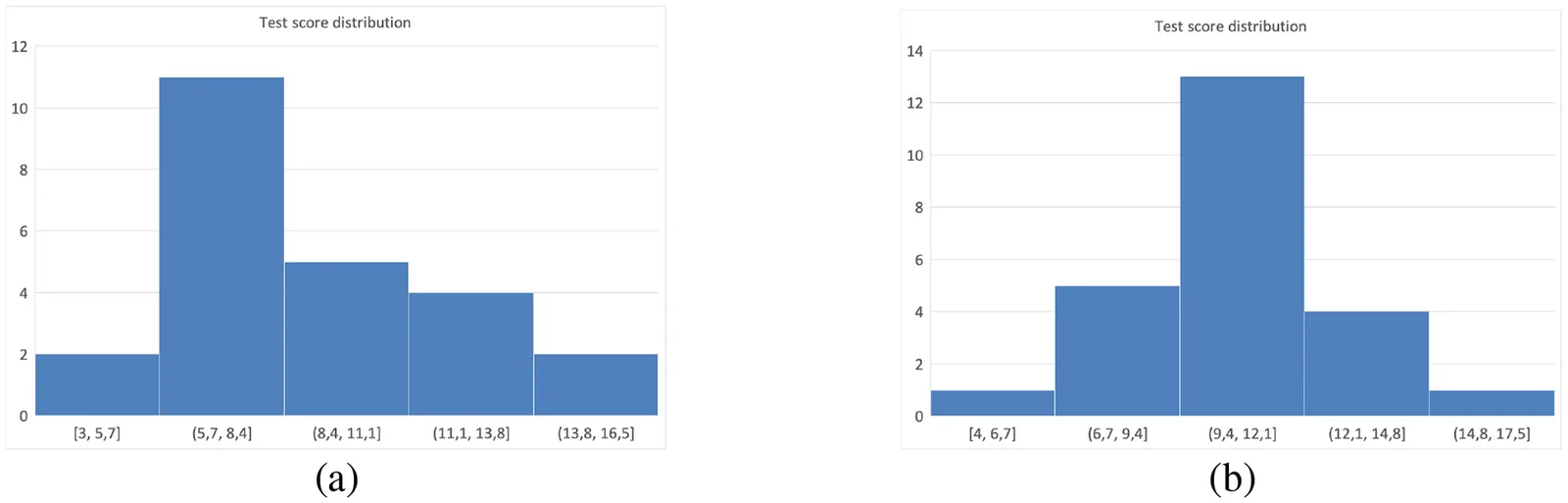
Distributions of test scores among participants for control **(a)** and experimental **(b)** group.

Figure 6 reports test score distributions for the control group (*M* = 8.92, *SD* = 3.11) and for the experimental group (*M* = 10.63, *SD* = 2.39).

Taken together, these results support the first two research hypotheses by demonstrating that the integration of a digital avatar can significantly enhance knowledge acquisition and motivation compared to traditional self-directed learning approaches, while the data suggest these benefits are not conditioned by gender.

Table 2 summarizes all the main statistical results for all the dependent variables, reporting for each experimental outcome *F, p, partial* *η*^2^, *Cohen's f*, λ and *Post-hoc power*.

**Table 2:** Summary of statistical results.

Outcome	*F*	*p*	partial *η*^2^	Cohen's *f*	λ	*Post-hoc* power
Knowledge acquisition	4.657	.036	.096	.325	5.080	.597
Post-test academic motivation, SDI	4.022	.051	.084	.302	4.388	.535
Change in academic motivation, ΔSDI	9.221	.004	.173	.458	10.059	.873
Cognitive load, total	0.163	.688	.004	.061	0.178	.070
Intrinsic cognitive load	1.667	.203	.036	.195	1.818	.261
Extraneous cognitive load	0.379	.541	.009	.093	0.414	.096

## 4 Discussion

The present study investigated the impact of AI-powered digital avatars on three key dimensions of the learning experience: knowledge acquisition, academic motivation, and perceived cognitive load. Participants who studied with the support of a human-like AI avatar were compared to peers who performed an equivalent learning task using autonomous internet search. The results suggest that the avatar-based learning condition was more effective in promoting educational outcomes, supporting the study's first and second hypotheses. However, no statistically significant differences emerged regarding perceived cognitive load, suggesting only partial support for the proposed hypotheses.

The main effect of instructional condition was statistically significant, indicating that students who interacted with the AI avatar not only acquired more knowledge but also experienced higher motivation than those in the control condition. These findings align with recent literature on virtual pedagogical agents, particularly those that integrate multi-modal and anthropomorphic features, which are thought to enhance engagement and support deeper processing of information (Dai et al., 2024; Mayer, 2014). The avatar's ability to deliver content in a structured, expressive, and context-sensitive manner likely functioned as an external cognitive scaffold, reducing ambiguity and enabling more focused attention on relevant learning targets.

Moreover, while the main effect of gender was not significant, the absence of an interaction effect between instructional condition and gender is equally noteworthy. It suggests that the observed educational advantages of avatar-based learning were broadly consistent across male and female participants, enhancing the ecological validity of the findings and indicating that such technologies could be effectively scaled across diverse learner populations without a loss in pedagogical efficacy.

The novelty of this study lies in its empirical examination of next-generation AI-powered digital avatars as instructional agents and their comparison with conventional self-directed online learning. Unlike prior studies focused mainly on rule-based virtual tutors or traditional pedagogical agents, this research contributes evidence regarding the educational potential of advanced AI-driven avatars in supporting both cognitive and motivational dimensions of learning.

However, some caution is warranted when interpreting these findings. Although the overall trend favored the avatar condition, the effect on perceived cognitive load did not reach statistical significance in the current analysis. This may be due to ceiling effects or the short duration of the intervention, which might have limited the observable impact on complex metacognitive processes such as load regulation. These results indicate that AI-avatar-supported instruction may be particularly beneficial for facilitating learning performance, although further investigation is needed to determine its broader cognitive effects. It is also possible that the novelty of the avatar may have introduced a transient form of extraneous load, masking its longer-term cognitive benefits. Future research using longitudinal designs or eye-tracking methods could help disentangle these competing mechanisms.

### 4.1 Limitations and considerations

In addition, it is important to note some limitations: The study design does not permit firm conclusions regarding the specific causal mechanisms underlying observed differences in learning outcomes. In particular, it cannot be determined whether the effects were attributable to anthropomorphic characteristics of the avatar, increased interactivity, structured guidance, novelty effects, or other features of the learning environment. Furthermore, the absence of statistically significant effects on perceived cognitive load suggests that the educational impact of AI-avatar systems may not be uniform across all learning-related dimensions. Accordingly, the present findings should be interpreted as preliminary evidence of potential educational benefits rather than definitive proof of the superiority of anthropomorphic AI-avatar design. Moreover, several participant-related factors should also be considered as potential confounders. Although participants were randomly assigned to experimental conditions, the sample was relatively small, geographically concentrated, and predominantly composed of individuals from a similar national background, with a substantial proportion originating from Iran, all conditions which may have resulted in bias toward the instructional content and / or the experimental procedure, due to cultural and or background related similarities. Because of the nature of the intervention, neither the participants nor the researchers supervising the experimental sessions could be blinded to group allocation. Consequently, expectancy effects cannot be ruled out and should be considered when interpreting the findings. In addition, variability in English language proficiency and heterogeneous educational backgrounds may have influenced participants' engagement with the learning materials and interaction with the AI-avatar system, as a lower performance score could be related to a lower language proficiency rather than a less effective learning method. Differences in digital literacy, prior familiarity with AI-based technologies, and language comprehension could potentially affect knowledge acquisition and learning experiences, is spite of the lack of test score correlation with prior specific knowledge of the subject being studied [see Figures 4c, 5c]. Due to the sample size and exploratory nature of the study, these variables were not included as statistical covariates. Future research should incorporate larger and more diverse samples and consider controlling for language proficiency, digital literacy, prior AI exposure, and educational background to better isolate the effects of AI-avatar-supported learning. Another valuable direction for future research could involve comparing different types of avatars (e.g., static, text-based, embodied 3D models) to isolate the specific features that contribute most to learning effectiveness. Given the exploratory nature of the study, baseline equivalence in domain-specific knowledge was not assessed prior to the intervention. Future research should incorporate pre-intervention knowledge assessments to establish baseline equivalence and strengthen causal inference.

Despite these limitations, the findings contribute meaningfully to the growing body of evidence supporting the use of intelligent pedagogical agents in digital education. As AI technologies continue to evolve, their application in the educational sector holds great promise—not only for enhancing cognitive performance but also for fostering motivation, personalizing instruction, and promoting broader access to high-quality learning experiences. These results advocate for a thoughtful and evidence-based integration of AI avatars into educational practice, supported by ongoing research and careful attention to issues of usability, equity, and ethical deployment.

## Data Availability

The raw data supporting the conclusions of this article will be made available by the authors, without undue reservation.

## References

[B1] Acosta Echavarria A. A. Mejía Toro W. A. González Uribe A. M. (2022). IQ and academic performance in a group of first semester psychology students. Pensamiento Americano 15, 153–167. doi: 10.21803/penamer.15.29.450

[B2] Alam A. Mohanty A. (2023). “Facial analytics or virtual avatars: competencies and design considerations for student-teacher interaction in AI-powered online education for effective classroom engagement,” in Communication, Networks and Computing, eds., R. S. Tomar (Cham: Springer). doi: 10.1007/978-3-031-43145-6_21

[B3] Apoki U. C. Hussein A. M. A. Al-Chalabi H. K. M. Badica C. Mocanu M. L. (2022). The role of pedagogical agents in personalised adaptive learning: a review. Sustainability 14:6442. doi: 10.3390/su14116442

[B4] Azevedo R. (2005). Computer environments as metacognitive tools for enhancing learning. Educ. Psychol. 40, 193–197. doi: 10.1207/s15326985ep4004_1

[B5] Balzer L. B. Petersen M. L. van der Laan M. J. (2014). Adaptive pair-matching in randomized trials with unbiased and efficient effect estimation. Stat. Med. 34, 999–1011. doi: 10.1002/sim.6380 25421503 PMC4318754

[B6] Bandura A. (1986). Social Foundations of Thought and Action: A Social Cognitive Theory. Englewood Cliffs, NJ: Prentice-Hall.

[B7] Beege M. Schroeder N. L. Heidig S. Schneider S. (2023). The instructor presence effect and its moderators in instructional videos: a series of meta-analyses. Educ. Res. Rev. 47:101101. doi: 10.1016/j.edurev.2023.100564

[B8] Castro-Alonso J. Wong R. M. Adesope O. O. Paas F. (2021). Effectiveness of multimedia pedagogical agents predicted by diverse theories: a meta-analysis. Educ. Psychol. Rev. 33, 989–1015. doi: 10.1007/s10648-020-09587-1

[B9] Chen B. Zhang Y. Wang S. Gan X. Wang G. Wang N. (2025). “E-education software integrating AI: Aimed at enhancing learner engagement, in *Procedding of 2nd International Conference on Intelligent Computing and Robotics (ICICR)* (Dalian: IEEE), 199–207. doi: 10.1109/ICICR65456.2025.00042

[B10] Dai C.-P. Ke F. (2022). Educational applications of artificial intelligence in simulation-based learning: a systematic mapping review. Comput. Educ. Artif. Intell. 3:100087. doi: 10.1016/j.caeai.2022.100087

[B11] Dai C.-P. Ke F. Pan Y. Moon J. Liu Z. (2024). Effects of artificial intelligence-powered virtual agents on learning outcomes in computer-based simulations: a meta-analysis. Educ. Psychol. Rev. 36:31. doi: 10.1007/s10648-024-09855-4

[B12] Dai L. Jung M. M. Postma M. Louwerse M. M. (2022). A systematic review of pedagogical agent research: Similarities, differences and unexplored aspects. Comput. Educ. 190:104607. doi: 10.1016/j.compedu.2022.104607

[B13] Dargue N. Sweller N. Jones M. P. (2019). When our hands help us understand: a meta-analysis into the effects of gesture on comprehension. Psychol. Bull. 145, 765–784. doi: 10.1037/bul0000202 31219263

[B14] Deci E. L. Ryan R. M. (1985). Intrinsic Motivation and Self-Determination in Human Behavior. New York, NY: Plenum. doi: 10.1007/978-1-4899-2271-7

[B15] Fabio R. A. Plebe A. Suriano R. (2025). AI-based chatbot interactions and critical thinking skills: an exploratory study. Curr. Psychol. 44, 8082–8095. doi: 10.1007/s12144-024-06795-8

[B16] Fink M. C. Robinson S. A. Ertl B. (2024). AI-based avatars are changing the way we learn and teach: benefits and challenges. Front. Educ. 9:1416307. doi: 10.3389/feduc.2024.1416307

[B17] Herbert C. Dołżycka J. D. (2024). “Teaching online with an artificial pedagogical agent as a teacher and visual avatars for self-other representation of the learners. Front. Educ. 9:1416033. doi: 10.3389/feduc.2024.1416033

[B18] Hicks S. A. (2024). Paper No.: 3238. The importance of self-regulation and motivation in online learning environments. (master's thesis). Fort Hays State University, Hays, KS, United states.

[B19] Ishak M. Oderinde I. Ahmad S. (2025). The role of metacognitive strategies in enhancing learning outcomes and educational efficiency: a systematic review. Int. J. Acad. Res. Bus. Soc. Sci. 15, 81–101. doi: 10.6007/IJARBSS/v15-i4/24964

[B20] Johnson W. L. Lester J. C. (2016). Face-to-face interaction with pedagogical agents, twenty years later. Int. J. Artif. Intell. Educ. 26, 25–36. doi: 10.1007/s40593-015-0065-9

[B21] Krell M. (2015). Evaluating an Instrument to Measure Mental Load and Mental Effort Using Item Response Theory. Berlin: Science Education Review Letters.

[B22] Krell M. Xu K. M. Rey G. D. Paas F. (2022). Recent approaches for assessing cognitive load from a validity perspective. Front. Educ. 6:838422. doi: 10.3389/978-2-88974-520-3

[B23] Lacković N. Olteanu A. (2026). Relational and Multimodal Higher Education: Digital, Social and Environmental Perspectives. Oxfordshire: Routledge.

[B24] Lane H. C. Schroeder N. L. (2022). Pedagogical Agents in Educational Technology: A Review of the Literature. New York, NY: Educational Technology Research and Development.

[B25] Li K. Pengbo S. (2024). Effectiveness of facial anthropomorphism design for improving multimedia learning outcomes: systematic review and meta-analysis. Smart Learn. Environ. 11:42. doi: 10.1186/s40561-024-00332-7

[B26] Li W. Kuang Z. Leng X. Mayer R. E. Wang F. (2024). Role of gesturing onscreen instructors in video lectures: a set of three-level meta-analyses on the embodiment effect. Educ. Psychol. Rev. 36:67. doi: 10.1007/s10648-024-09910-0

[B27] Li W. Wang F. Mayer R. E. (2023). How to guide learners' processing of multimedia lessons with pedagogical agents. Learn. Instruct. 84:101729. doi: 10.1016/j.learninstruc.2022.101729

[B28] Mayer R. E. (2014). “Principles based on social cues in multimedia learning: Personalization, voice, image, and embodiment,” in The Cambridge Handbook of Multimedia Learning, 2nd Edn., eds. R. E. Mayer (New York, NY: Cambridge University Press), 345–368. doi: 10.1017/CBO9781139547369.017

[B29] Mohammad Yusoff M. D. Yusof A. M. Mohd Anuar A. A. Akma Jamaludin N. S. Kamal Ruzzaman M. S. (2024). Effects of pedagogical agent on learner's cognitive load and intrinsic motivation: a systematic literature review. Pak. J. Life Soc. Sci. 22, 7316–7327. doi: 10.57239/PJLSS-2024-22.2.00552

[B30] Netland T. von Dzengelevski O. Tesch K. Kwasnitschka D. (2025). Comparing human-made and AI-generated teaching videos: an experimental study on learning effects. Comput. Educ. 224:105164. doi: 10.1016/j.compedu.2024.105164

[B31] Papakostas C. Troussas C. Krouska A. Sgouropoulou C. (2025). NAMI: a neuro-adaptive multimodal architecture for wearable human–computer interaction. Multimodal Technol. Interact. 9:108. doi: 10.3390/mti9100108.

[B32] Murphy P. Foley J. Mole J. Van Harskamp N. Cipolotti L. (2023). Lifespan normative data (18-89 years) for Raven's advanced progressive matrices set I. J. Neuropsychol. 17, 417-429. doi: 10.1111/jnp.12308 36808478

[B33] Raven J. (2000). The Raven's progressive matrices: change and stability over culture and time. Cogn. Psychol. 41, 1–48. doi: 10.1006/cogp.1999.0735 10945921

[B34] Raven J. C. (1986). Coloured Progressive Matrices, Sets A, A_B, B. London: H. K. Lewis and Co. Ltd.

[B35] Schinka J. A. LaLone L. (1997). MMPI-2 norms: comparisons with a census-matched subsample. Psychol. Assess. 9, 307–311. doi: 10.1037/1040-3590.9.3.307

[B36] Shehzad N. Nomaan S. Charles T. (2026). “A systematic review of AI's effect on students' mental health and well-being,” in Implications for Students' Mental Health in the Digital Age: AI and Cyber Behavior, ed. A. Shomotova, and A. ElSayary (Hershey, PA: IGI Global), 105–138. doi: 10.4018/979-8-3373-4222-1.ch005

[B37] Sui C. J. Yen M. H. Chang C. Y. (2024). Investigating effects of perceived technology-enhanced environment on self-regulated learning. Educ. Inf. Technol. 29, 161–183. doi: 10.1007/s10639-023-12270-x

[B38] Sun S. Hara A. Johnstone L. Hallmark B. Watkins J. C. Thomson C. A. (2024). Optimal pair matching combined with machine learning predicts a significant reduction in myocardial infarction risk. Nutrients 16:2933. doi: 10.3390/nu16172933 39275249 PMC11397525

[B39] Suriano R. Fabio R. A. Plebe A. (2025). “Theory of mind and social anxiety in emotional attachment to AI chatbots in individuals with autistic traits,” in Proceedings of the Annual Meeting of the Cognitive Science Society, vol. 47, eds. Barner, N. R. Bramley, A. Ruggeri and C. M. Walker (Oakland, CA: eScholarship), 3185–3192.

[B40] Sweller J. (2011). “Cognitive load theory,” in Psychology of Learning and Motivation, eds., J. P. Mestre, and B. H. Ross, vol. 55 (Cambridge, MA: Academic Press), 37–76. doi: 10.1016/B978-0-12-387691-1.00002-8

[B41] Teng Y. Wang X. (2024). Full caption, english proficiency and their relationships with behavioral, cognitive and emotional student engagement in chinese efl college content-based instruction video learning. Educ. Inf. Technol. 29, 861–880. doi: 10.1007/s10639-023-12342-y

[B42] Vallerand R. J. Reid G. (1984). On the causal effects of perceived competence on intrinsic motivation: a test of cognitive evaluation theory. J. Sport Exerc. Psychol. 6, 94–102. doi: 10.1123/jsp.6.1.94

[B43] Wang F. Li W. Mayer R. E. Liu H. (2018). Animated pedagogical agents as aids in multimedia learning: Effects on eye-fixations during learning and learning outcomes. J. Educ. Psychol. 110, 250–268. doi: 10.1037/edu0000222

[B44] Wu W. Mo Z. Yu W. Cheng Y. Zhang T. Huang J. (2024). “Generalizable geometry-aware human radiance modeling from multi-view images,” in Pattern Recognition and Computer Vision, eds. Z. Lin, M. M. Cheng, R. He, K. Ubul, W. Silamu, and H. Zha (Singapore: Springer), 95–109. doi: 10.1007/978-981-97-8508-7_7

